# A real-world economic analysis of biologic therapies for moderate-to-severe plaque psoriasis in Italy: results of the CANOVA observational longitudinal study

**DOI:** 10.1186/s12913-021-06866-7

**Published:** 2021-09-06

**Authors:** Emanuela Zagni, Luca Bianchi, Gabriella Fabbrocini, Salvatore Corrao, Annamaria Offidani, Luca Stingeni, Antonio Costanzo, Giovanni Pellacani, Ketty Peris, Federico Bardazzi, Giuseppe Argenziano, Silvana Ruffolo, Paolo Dapavo, Carlo Carrera, Maria Concetta Fargnoli, Aurora Parodi, Marco Romanelli, Piergiorgio Malagoli, Marina Talamonti, Matteo Megna, Massimo Raspanti, Matteo Paolinelli, Katharina Hansel, Alessandra Narcisi, Andrea Conti, Clara De Simone, Marco Adriano Chessa, Alina De Rosa, Eugenio Provenzano, Michela Ortoncelli, Chiara Moltrasio, Rosaria Fidanza, Martina Burlando, Annalisa Tonini, Francesca Maria Gaiani, Lucia Simoni, Alessandro Zullo, Martina Fiocchi, Delia Colombo

**Affiliations:** 1 Novartis Farma S.p.A, Largo Umberto Boccioni, 1, 21040 Origgio, Varese Italy; 2 Policlinico Tor Vergata, Rome, Italy; 3 Section of Dermatology, Department of Clinical Medicine and Surgery, University of Naples Federico II, Naples, Italy; 4 ARNAS Civico, Palermo, Italy; 5 Dermatology Unit, Department of Clinical and Molecular Sciences, Polytechnic Marche University, Ancona, Italy; 6 Dermatology Section, Department of Medicine, University of Perugia, Perugia, Italy; 7 IRCCS Istituto Clinico Humanitas, Rozzano, Italy; 8 Dermatology Clinic, Department of Clinical Internal, Anesthesiological and Cardiovascular Sciences, La Sapienza University of Rome, Rome, Italy; 9 Fondazione Policlinico Universitario A. Gemelli-IRCCS and Università Cattolica, Rome, Italy; 10 Dermatology, IRCCS Policlinico di S Orsola Department of Experimental, Diagnostic and Specialty Medicine (DIMES) Alma Mater Studiorum University of Bologna, Bologna, Italy; 11 Dermatology Unit, University of Campania, Naples, Italy; 12 A.O. Cosenza Ospedale SS Annunziata, Cosenza, Italy; 13 A.O.U. Città della Salute e della Scienza PO Molinette, Turin, Italy; 14 Fondazione IRCCS Ca’Granda Ospedale Maggiore Policlinico, Milan, Italy; 15 Dermatology, Department of Biotechnological and Applied Clinical Sciences, University of L’Aquila, L’Aquila, Italy; 16 Clinica Dermatologica DiSSal Università di Genova/Ospedale-Policlinico San Martino IRCCS, Genoa, Italy; 17 U.O. Dermatologia Universitaria - Azienda Ospedaliero Universitaria Pisana, Pisa, Italy; 18 IRCCS Policlinico San Donato, San Donato Milanese, Italy; 19 A.O.U. Federico II, Naples, Italy; 20 A.O.U. Policlinico, Modena, Italy; 21 MediNeos Observational Research, Modena, Italy

**Keywords:** Biologic, Secukinumab, Adalimumab, Ustekinumab, Ixekizumab, Costs, Cost per responder, Response rate, Real-world

## Abstract

**Background:**

Psoriasis is a chronic immune-mediated inflammatory skin disease which can also involve joints. It is often associated with burdensome comorbidities which negatively impact prognosis and quality of life (QoL). Biologic agents have been shown to be effective in controlling disease progression, but their use is associated with higher costs compared with traditional systemic treatments. The economic analysis of the CANOVA (EffeCtiveness of biologic treAtmeNts for plaque psOriasis in Italy: an obserVAtional longitudinal study of real-life clinical practice) study aims to assess the costs and cost-effectiveness of biologics in a real-world context in Italy.

**Methods:**

The annualised overall direct costs of moderate-to-severe plaque psoriasis management, the annualised cost of biologic drugs and the cost per responder in the Italian National Health System perspective were assessed. More specifically, the cost per response and cost per sustained response of the most prescribed biologic therapies for the treatment of moderate-to-severe plaque psoriasis within the CANOVA study were assessed using the Psoriasis Area Severity Index (PASI) at several score levels (75, 90 and 100%).

**Results:**

The most frequently used biologic therapies for plaque psoriasis were secukinumab, ustekinumab, adalimumab originator, and ixekizumab. Cost of biologics was the driver of expenditure, accounting for about 98% of total costs. Adalimumab originator was the biologic with the lowest cost per responder ratio (range: €7848 - €31,378), followed by secukinumab (range: €9015 - €33,419). Ustekinumab (range: €11,689 – €39,280) and ixekizumab (range: €11,092 – €34,289) ranked respectively third and fourth, in terms of cost-effectiveness ratio. As concerns the cost per sustained response analysis, secukinumab showed the lowest value observed (€21,375) over the other options, because of its high response rate (86% vs. 60–80%), which was achieved early in time.

**Conclusion:**

Biologic therapy is a valuable asset for the treatment of moderate-to-severe plaque psoriasis. Concomitant assessment of treatment costs against the expected therapeutic response over time can provide physicians and payers additional insights which can complement the traditional risk-benefit profile assessment and drive treatment decisions.

**Supplementary Information:**

The online version contains supplementary material available at 10.1186/s12913-021-06866-7.

## Background

Psoriasis is a chronic immune-mediated inflammatory skin disease which can also involve joints. It is often associated with burdensome comorbidities (arthritis, cardiovascular diseases, metabolic syndrome, and inflammatory bowel disease) which negatively impact prognosis and QoL, even when the affected body surface area (BSA) is relatively limited [1–3]. In addition to physical pain, psoriasis causes social and psychological burden: social exclusion, discrimination, and stigma can be devastating for patients, who suffer from burdensome depression, as demonstrated by a number of studies [2, 4–7]. Severity of psoriasis is related to several different aspects of this disease, including extent of psoriasis, location of lesions, degree of inflammation, responsiveness to treatment and impact on QoL, although no validated categories of severity are internationally recognised [8].

Psoriasis can occur at any age, but it is more common between 50 and 69 years of age [2]. It is considered equally prevalent in both sexes, although some studies reporting prevalence by sex indicated that psoriasis is more common in men. The worldwide prevalence of psoriasis is highly variable, ranging between 0.91% in the United States and 11.43% in Norway [2]. In Italy, the estimated psoriasis incidence is 2.30–3.21 cases per 1000 per person per year [9], with a prevalence range of 1.8–3.1% [10]. Plaque psoriasis, characterised by well-defined round or oval plaques that differ in size and often coalesce, is the most prevalent clinical type of psoriasis, affecting between 58 and 97% of all patients [2], with about 20–30% of them suffering from a moderate or severe condition [11]. As regards Italy, 2.8% of the entire population suffer from plaque psoriasis [12].

The severity of chronic plaque psoriasis is generally assessed with different tools. Typically, efficacy of psoriasis treatments in reducing the burden of disease is measured (in clinical trials) in terms of proportion of patients achieving > 75% reduction in their baseline Psoriasis Area and of Severity Index score (PASI 75) after 12 weeks of therapy and later in time.

The management of plaque psoriasis is highly variable, and guidelines are published at national as well as European level [13]. Typically, patients with severe plaque psoriasis are considered for systemic therapy and/or phototherapy. Conventional systemic agents include acitretin, cyclosporine, and methotrexate. During the last decade, the development of biologic agents, such as infliximab, etanercept, adalimumab, ustekinumab, secukinumab, and ixekizumab, has changed the therapeutic landscape of moderate-to-severe plaque psoriasis [14–18]. Treatment regimens for psoriasis patients should be tailored to meet their specific needs based on disease severity, impact on quality of life, response to previous therapies, and any comorbidities, such as hepatitis, malignancy, obesity, and cardiovascular diseases [19, 20]. Biologic agents have shown greater efficacy and fewer adverse events compared to traditional therapies in clinical trials, but observational, real-life studies are needed to determine whether these effects persist during long-term therapy in clinical practice [21]. Biologic agents have clinically proven efficacy, but their use is associated with much higher costs compared with traditional systemic treatments [22]. Therefore, there is a great need to evaluate efficiency and costs of healthcare interventions in real practice. At the time of launch of a new treatment, economic evidence is tested through models (cost-effectiveness, budget impact), assuming that the outcomes observed in clinical trials are achieved in clinical practice as well. Also, evidence of long-term effects is rarely available at the launch of a new treatment; therefore, simulations are used to predict “lifetime” costs and clinical consequences of treatment use. Finally, health technology assessment agencies, and pricing and reimbursement institutions are interested in evaluating the economic implications of the adoption of new technology in their respective countries [23]. The use of observational research to collect economic evidence would overcome the typical limitations of these economic models, and inform decision makers with “real-world” cost-effectiveness indicators [24].

Given the chronic nature of plaque psoriasis and the considerable amount of direct costs attributable to it (pharmacological treatment, hospitalisations, monitoring, etc.) [11, 25, 26], analysis of real-world data is important to assess the economic burden of the disease [27].

The CANOVA was an observational study designed to provide real-world evidence of the effectiveness of biologic treatments for plaque psoriasis in Italy [27]. The analysis presented in this paper refers to the economic secondary endpoints evaluated in the context of the CANOVA study. Finally, the annualised management and biologics costs for moderate-to-severe plaque psoriasis, as well as the cost per responder and cost per sustained responder ratios in the Italian National Health System perspective were assessed. More specifically, we analysed costs and cost-effectiveness of the most prescribed biologic therapies used in the treatment of moderate-to-severe plaque psoriasis within the CANOVA study.

## Methods

### Source of clinical data

#### Observational study design

The CANOVA study is a multicenter, non-interventional longitudinal study which involved both the use of primary data (registered during the visits in the CANOVA study) and secondary data (collected from medical records of each participating patient). The study was conducted in 17 Italian hospital dermatology clinics, which enrolled *N* = 727 patients. Patients aged ≥18 years, with moderate-to-severe plaque psoriasis, having provided written informed consent before data collection and having initiated biologic treatments between 24 weeks and 24 months before enrolment visit (retrospective period), were included. In order to avoid potential selection bias, patients who interrupted treatment before enrolment could also be included. All treatments had been administered according to current clinical practice. Pregnant or breast-feeding women, patients receiving or having received biologic treatments for psoriasis as part of a clinical trial, or having no available information about biologic treatments and response were excluded. Patients were withdrawn from the study in case of withdrawal of informed consent or privacy form, death, loss to follow-up, inclusion in a clinical trial involving treatment with biologic agents for psoriasis, or pregnancy. The study design ensured a total length of observation (retrospective + prospective) of at least 12 months for each patient (except for withdrawals). For instance: i) patients enrolled 6 months (24 weeks) after starting a biologic therapy line were prospectively observed for 6 months, and underwent 2 visits (enrolment + 6-month follow-up visit), for a total observation period of 12 months; ii) patients enrolled 24 months after starting a biologic therapy line were prospectively observed for 6 months, and underwent 2 visits (enrolment + 6-month follow-up visit), for a total observation period of 30 months. The Patient’s scheme is provided in Additional File 1. Enrollment lasted from 24 April 2018 (First Patient In) to 26 February 2019 (Last Patient In). End date of data collection (Last Patient Last Visit) was 31 October 2019 [27].

#### Treatments

In the study, all eligible patients (*N* = 669, overall group from now on) were treated with at least one of the following biologic therapies: secukinumab (Cosentyx), ustekinumab (Stelara), adalimumab originator (Humira), adalimumab biosimilar (Amgevita), adalimumab biosimilar (Imraldi), ixekizumab (Taltz), certolizumab (Cimzia), etanercept (Enbrel), etanercept biosimilar (Benepali), golimumab (Simponi).

For subgroup analyses, the “reference biologic therapy”, identified by the type of biologic drug (active substance and originator / biosimilar features), was defined as the biologic therapy ongoing at enrollment (or the most recently interrupted treatment with respect to enrollment, in case of no ongoing biologic therapy) [27].

#### Response criteria

The primary objective of the CANOVA study was to assess effectiveness of biologic therapies in the treatment of moderate-to-severe plaque psoriasis [27]. The Psoriasis Area Severity Index (PASI) is one of the most largely used psoriasis rating scales in clinical trials [28–30]. PASI is a measure of the average severity of psoriasis skin clinical signs weighted by the affected area in four body regions: head, upper limbs, trunk, and lower limbs. The PASI index, with a range between 0 and 72, is obtained by summing up the severity score of each body region, [28].

According to the European consensus of treatment goals, treatment success is defined as a 75% or more reduction in the Psoriasis Area and Severity Index score (PASI 75) which allows for treatment continuation [31]. In the context of the economic analyses presented in this paper, PASI 75, PASI 90, and PASI 100 in respect of the score evaluated at the initiation of the reference biologic treatment line are considered to estimate the cost-effectiveness of the biologic treatments. In the evaluation of response according to PASI, patients who had available information about clinical response/no response at week 16, 24, and 52 were considered. An evaluation of the clinical response at time point *t* (*t* = 16, 24, 52 weeks) was possible for patients with available PASI score at the start of biologic therapy and at time point *t*. In case of therapy discontinuation before time point *t* due to lack of efficacy of biologic therapy, the patient was included in the evaluation of the response (to avoid potential selection bias) and considered as non responder at time point *t*. In this case, the PASI scores assessed after therapy interruption were not taken into consideration. In case, instead, a patient discontinued therapy before time point *t*, due to reasons different from lack of efficacy (such as side effects, patient decision, or lack of compliance), and had an available PASI score at time point t recorded within 4 weeks after therapy discontinuation, then the patient was included in the evaluation of response and the PASI score was taken into consideration.

### Source of economic data

#### Resource consumption

The following resource consumption data were collected to determine costs of moderate-to-severe plaque psoriasis: i) pharmacological biologic treatments received during the study period, settings of administration (inpatient, ambulatory outpatient, home), frequency of treatment, duration of treatment; ii) pharmacological / topical concomitant (adjunctive) moderate-to-severe plaque psoriasis treatments (topical corticosteroids, calcipotriol, methotrexate); iii) biologic treatment-related adverse events (drugs prescribed, follow-up visits, procedures, hospitalisations to manage the adverse events (AEs); iv) treatment follow-up/monitoring during the study period (specialist visits, laboratory examinations, diagnostic examinations); v) other interventions during the study period (hospitalisations due to disease progression / worsening, phototherapy).

#### Costs

The economic analyses were conducted adopting the Italian national healthcare service (NHS, or SSN in Italian) perspective: therefore, only direct costs sustained by the Italian SSN were considered and collected.

Ex-manufacturer acquisition costs of therapies and tariffs for outpatient and inpatient services were retrieved from national databases [32, 33].

For systemic biologic therapies, the cost was obtained by multiplying the unit cost by the estimated total quantity of drug received by the patient during the observation period, taking into consideration treatment duration and posology as declared and prescribed by the treating physician.

The costs of topical therapies and of other pharmacological / topical therapies for moderate-to-severe plaque psoriasis, as well as of other relevant concomitant medications to manage adverse events related to biologic treatments, were obtained by multiplying the unit cost by the estimated total quantity of drug received by the patient during the observation period, according the treatment duration declared by the physician; posology was estimated based on the indications in the Summary of Product Characteristics of each drug.

Phototherapy costs were obtained by multiplying the unit cost by the total number of sessions received.

Charges for Emergency department (ED) accesses, and inpatient hospitalisations were based on DRG (diagnosis related group) codes. General practitioner (GP) visit costs for moderate-to-severe plaque psoriasis were allotted according to Garattini et al. [34] (Cost inflated from January 2003 to October 2019 [35]). The costs of specialist outpatient visits for moderate-to-severe plaque psoriasis were assigned according to Italian Ministry of Health, outpatient, and hospital tariffs, respectively [32, 36].

Test/procedures/instrumental examination costs were assigned according to Italian Ministry of Health, outpatient tariffs [32].

### Economic analysis

No formal statistical hypotheses were set. Descriptive analyses were performed (comparison between treatments was not in the scope of the study).

The economic assessment consisted of three different analyses in the Italian National Health System perspective: i) moderate-to-severe plaque psoriasis annualised management and biologics costs analysis; ii) cost per response analysis, iii) cost per sustained response analysis.

The annualised cost of moderate-to-severe plaque psoriasis patients was calculated, for each patient, as the sum of the costs of therapies and healthcare services since initiation of the reference biologic therapy divided by the observation window from the start of the reference biologic therapy (in years). Each patient then had a specific annual cost of management: such values returned the estimated annualised cost of patient management. The cost per responder ratio was calculated to estimate cost-effectiveness of the reference biologic therapies and it was defined as the amount of investment required to successfully treat one patient, according to PASI 75, PASI 90, and PASI 100 response criteria. The cost per responder ratio at 16 weeks (tolerance window: 10 to 20 weeks), 24 weeks (tolerance window: 20 to 30 weeks), and 52 weeks (tolerance window: 40 to 64 weeks) was calculated as the sum of individual costs, sustained over the first 16/24/52 weeks from the start of the reference biologic therapy (considering all sources of costs sustained over the first 16/24/52 weeks from the start of the reference biologic therapy and prior to subsequent biologic line, if any) divided by the number of patients achieving clinical response (in terms of PASI 75/90/100) at the respective time point, using the following equation:

$$ \mathrm{Cost}\ \mathrm{per}\ \mathrm{responder}\ \mathrm{with}\ \mathrm{treatment}\ x\ \mathrm{at}\ \mathrm{time}\ \mathrm{point}\ t=\frac{\mathrm{Total}\ \mathrm{cost}\ \mathrm{of}\ \mathrm{patients}\ \mathrm{treated}\ \mathrm{with}\ \mathrm{treatment}\ x\ \left(\mathrm{from}\ \mathrm{the}\ \mathrm{start}\ \mathrm{of}\ \mathrm{the}\ \mathrm{the}\mathrm{rapy}\ \mathrm{to}\ \mathrm{time}-\mathrm{point}\ t\right)\ }{\mathrm{Number}\ \mathrm{of}\ \mathrm{patients}\ \mathrm{achieving}\ \mathrm{clinical}\ \mathrm{response}\ \left(\mathrm{in}\ \mathrm{terms}\ \mathrm{of}\ \mathrm{PASI}\ 75/90/100\right)\ \mathrm{at}\ \mathrm{time}-\mathrm{point}\ t} $$

Where *x* = overall cohort, secukinumab, ustekinumab, adalimumab originator, ixekizumab, and *t* = 16, 24, 52 weeks.

Finally, a post-hoc analysis was carried out to measure the cost per sustained responder ratio, defined as the amount of investment required to achieve PASI 75 response both at 16 and at 52 weeks. The cost per sustained responder ratio at 52 weeks was calculated as the sum of individual costs during the first 52 weeks from the start of the reference biologic therapy (and prior to subsequent biologic line, if any), divided by the number of patients achieving clinical response (in terms of PASI 75) both at 16 and at 52 weeks, using the following equation:

$$ \mathrm{Cost}\ \mathrm{per}\ \mathrm{sustained}\ \mathrm{responder}\ \mathrm{with}\ \mathrm{treatment}\ x\ \mathrm{at}\ \mathrm{at}\ 52\ \mathrm{weeks}=\frac{\mathrm{Total}\ \mathrm{cost}\ \mathrm{of}\ \mathrm{patients}\ \mathrm{treated}\ \mathrm{with}\ \mathrm{treatment}\ x\ \left(\mathrm{from}\ \mathrm{the}\ \mathrm{start}\ \mathrm{of}\ \mathrm{the}\ \mathrm{the}\mathrm{rapy}\ \mathrm{to}\ \mathrm{week}\ 52\right)\ }{\mathrm{Number}\ \mathrm{of}\ \mathrm{patients}\ \mathrm{achieving}\ \mathrm{clinical}\ \mathrm{response}\ \left(\mathrm{in}\ \mathrm{terms}\ \mathrm{of}\ \mathrm{PASI}\ 75/90/100\right)\ \mathrm{both}\ \mathrm{at}\ 16\ \mathrm{and}\ \mathrm{at}\ 52\ \mathrm{weeks}} $$

Where *x* = overall cohort, secukinumab, ustekinumab, adalimumab originator, ixekizumab.

The economic analyses were carried out for the overall cohort and for the most numerous reference biologic therapy subgroups contributing at least to 10% of the sample size within the CANOVA study (i.e. Secukinumab, Ustekinumab, Adalimumab originator, Ixekizumab).

## Results

### Participants and subgroups

A total of *N* = 727 patients were enrolled in the CANOVA study. Among these, *N* = 669 patients (92.0% of enrolled subjects) were eligible based on inclusion/exclusion criteria [27]. Considering only the biologic drug ongoing at the enrolment visit (or the most recently interrupted, if any), the most frequently used biologic medication for psoriasis in the eligible population was secukinumab (41.0%; *n* = 274), followed by ustekinumab (25.3%; *n* = 169), adalimumab originator (13.0%; *n* = 87), and ixekizumab (12.1%; *n* = 81). The complete list of biologics in use at enrolment is reported in Table 1.

**Table 1:** Biologic treatments details: type of treatments (Eligible patients)

Reference biologic treatment line	Eligible patients N = 669 n (%)
Secukinumab (Cosentyx)	274 (41.0%)
Ustekinumab (Stelara)	169 (25.3%)
Adalimumab (Humira)	87 (13.0%)
Ixekizumab (Taltz)	81 (12.1%)
Certolizumab	19 (2.8%)
Etanercept (Enbrel)	17 (2.5%)
Etanercept (Benepali)	13 (1.9%)
Adalimumab (Amgevita)	5 (0.7%)
Golimumab	3 (0.4%)
Adalimumab (Imraldi)	1 (0.1%)

Overall median treatment duration was 17.9 months (interquartile range (IQR) 13.8–23.2; N = 669). In the treatment subgroups, the median treatment duration ranged from 14.2 months (IQR 11.9–16.3; N = 81) for the ixekizumab subgroups to 20.2 months (IQR 15.1–25.0; *N* = 168) for the ustekinumab subgroups.

Considering the reference biologic therapy, naïve patients were 52.1% of the overall sample, while 33.8% received only one previous biologic line, and 14.1% received more than one previous biologic treatment. The proportion of naïve patients was similar in the secukinumab, ustekinumab, and adalimumab groups, while biologic-naïve patients were about 35% of the ixekizumab group. Patients switching from one biologic treatment to another during the observation period were 6.4% of the overall population.

### Resource consumption

Table 2 provides a summary of resources used (specialist outpatient visits, tests/procedures/ instrumental examinations, GP visit, ED access, hospitalisation, and day-hospital visit) in the overall cohort, in terms of: i) proportion of patients using the resource at least once, during the observation period; ii) average rate of utilisation, per patient/year. As expected, almost all patients referred to specialists for moderate-to-severe plaque psoriasis management. Only 5.7% of the overall cohort of patients (*n* = 38) went to the GP for a plaque psoriasis-related issue, while nearly all of them (97.9%; *n* = 655) had at least one specialist outpatient visit during the observation period. The mean rate of specialist outpatient visits was 3.7 per patient/year (standard deviation (SD): 1.6; median: 3.6; IQR range: 2.7–4.7).

**Table 2:** Use of healthcare resources, by type (sorted by utilisation rates)

Resource	Number of patients using the resource (n)	Proportion of patients using the resource (%)	Frequency of resource use (n per patient/year)
Specialist outpatient visits	655	97.9%	3.7
Tests/Procedures/Instrumental examinations	516	77.1%	6.5
GP visits	38	5.7%	0.1
Day-hospital visits	26	3.9%	0.1
Hospitalisations	9	1.3%	NC
ED accesses	1	0.1%	NC

*ED: Emergency Department; GP: General Practitioner; NC: Not Calculated*

Use of tests, procedures and instrumental examinations was common in the observed cohort. The number of patients receiving at least one test, procedure, or instrumental examination for moderate-to-severe plaque psoriasis during the observation period was *n* = 516 (77.1%). On average, patients received 6.5 examination per year (SD: 6.1; median: 5.2; IQR: 0.8–10.6). The most common examinations (conducted in > 50% of the overall patients) were blood cell count, liver function test, kidney function test, glycemia.

The use of hospital resources (hospital admissions, ED accesses, duration of hospitalisation) during the observation period was extremely low, with only: i) 1.3% of the overall patients (*n* = 9) having at least one hospital admission; ii) 0.1% of the overall patients (*n* = 1) having one emergency department admission (due to a treatment-related adverse event); iii) 3.9% of the overall patients (*n* = 26) having at least one day-hospital visit. As a result, the economic impact of hospital care for the *n* = 669 patients followed-up during the observational period was negligible, compared with the cost of the pharmacological treatment with biologic drugs.

### Annualised costs

**Fig. 1:**
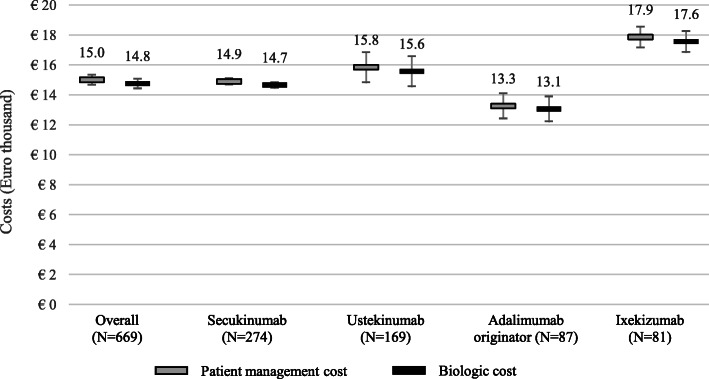
Annualised costs of patient management and biologic therapy (Mean, C.I. 95%)

Figure 1 shows the results of the moderate-to-severe plaque psoriasis management and biologic therapy cost analyses, respectively. On average, the annualised management cost of the overall cohort was €15,001 (interquartile range (IQR): €13,785 - €15,779). Almost all of this cost (98.34%) is attributable to biologic therapy (mean: €14,752; IQR range: €13,623 - €15,562). A certain variability of management costs was observed, by type of biologic drug. Adalimumab originator and ixekizumab were the subgroups with the lowest (€13,255) and highest (€17,858) annualised cost (Fig. 1), respectively. The variability of annualised costs was driven by the difference in the acquisition costs of the biologics. Same cost-ranking was observed in the management costs, with the adalimumab originator subgroup being the cheapest (€13,055) cohort of patients and ixekizumab the most expensive (€17,557).

### Cost per response analysis

The cost per response analysis was carried out on different numbers of patients at 16, 24, and 52 weeks. The study was mainly retrospective, possibly implying a certain heterogenicity in the consistency of the collected data. Not all patients had available complete data on clinical response at all study timepoints.

Cost-effectiveness of biologic therapies was evaluated by calculating the cost per responder ratio, defined as the cost required to successfully treat one patient (achieve clinical response). Calculation of cost per responder ratios depended on: i) timeframe (16 weeks, 24 weeks, 52 weeks); ii) type of outcome used to define “response” (PASI 75, PASI 90, PASI 100, sustained PASI 75 as post-hoc analysis).

Table 3 shows the results of the cost per response analysis, over the 16-, 24-, and 52-week period. At 16 weeks, secukinumab showed systematically the highest response rate through all the PASI criteria considered. Ixekizumab, ustekinumab, and adalimumab originator were ranked second, third, and fourth in the biologic response rate, considering PASI 75 and PASI 90 outcomes. The second, third, and fourth biologic in terms of PASI 100 response rate were ixekizumab, adalimumab originator, and ustekinumab.

**Table 3:** Results of the cost per response analysis at 16, 24, and 52 weeks

Outcome	Parameter	Week	Overall	Secukinumab	Ustekinumab	Adalimumab originator	Ixekizumab
PASI 75	Number of evaluated patients (N)	16	405	165	118	38	49
		24	380	154	105	46	38
		52	551	241	145	63	65
	Response rate (%)	16	86%	95%	80%	74%	90%
		24	90%	97%	87%	83%	89%
		52	91%	93%	94%	87%	91%
	Cost per responder ratio (€)	16	€9774	€9015	€11,689	€7848	€11,092
		24	€11,652	€10,982	€13,617	€10,069	€13,710
		52	€20,281	€19,932	€21,387	€18,491	€22,084
PASI 90	Number of evaluated patients (N)	16	391	162	113	36	46
		24	362	150	95	45	35
		52	540	238	140	62	63
	Response rate (%)	16	59%	64%	56%	50%	63%
		24	74%	84%	69%	67%	71%
		52	75%	77%	74%	71%	81%
	Cost per responder ratio (€)	16	€14,378	€13,395	€16,806	€11,560	€15,839
		24	€14,213	€12,742	€17,035	€12,477	€17,172
		52	€24,662	€23,978	€27,522	€22,755	€24,743
PASI 100	Number of evaluated patients (N)	16	421	172	123	38	52
		24	393	158	107	48	40
		52	564	243	150	66	65
	Response rate (%)	16	36%	42%	29%	32%	37%
		24	46%	51%	38%	56%	53%
		52	53%	55%	51%	52%	58%
	Cost per responder ratio (€)	16	€23,350	€20,079	€32,031	€18,312	€27,129
		24	€22,674	€20,857	€30,779	€14,784	€23,268
		52	€35,045	€33,419	€39,280	€31,378	€34,289

*PASI: Psoriasis Area Severity Index*

At 24 weeks, secukinumab showed the highest PASI 75 and PASI 90 response rate while the highest PASI 100 response rate was observed in adalimumab originator. The ixekizumab subgroup showed the highest PASI 75 and PASI 90 cost per responder ratio, followed by the ustekinumab, secukinumab, and adalimumab originator subgroups. The highest PASI 100 cost per responder ratio was observed in the ustekinumab subgroup, followed by the ixekizumab, secukinumab, and adalimumab originator subgroups.

At 52 weeks, ustekinumab showed the highest PASI 75 response rate whereas the highest PASI 90 and PASI 100 response rate were observed in ixekizumab. In terms of response rate, secukinumab always ranked second. At 52 weeks, the lowest and second lowest cost per responder ratio subgroups were systematically adalimumab originator and secukinumab. The ustekinumab subgroup showed the highest PASI 90 and PASI 100 cost per responder ratio while the most expensive PASI 75 cost per responder drug was ixekizumab.

### Cost per sustained response analysis (post-hoc analysis)

**Fig. 2:**
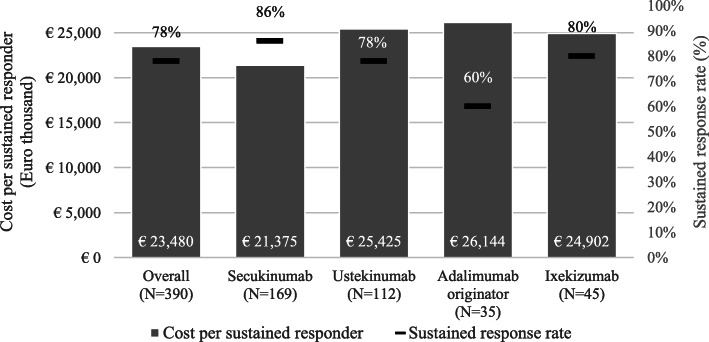
Results of the cost per sustained response analysis (based on PASI 75) at 52 weeks

An additional post-hoc cost per response analysis, based on PASI 75 sustained response at 52 weeks, was conducted to evaluate consistency of findings vs per-protocol analyses. Results are shown in Fig. 2. The average cost per sustained response according to PASI 75 for the overall cohort was €23,480. The cost per responder ratio varied by treatment subgroup, with the lowest value observed in the secukinumab subgroup (€21,375), and the highest value observed in the adalimumab originator subgroup (€26,144). The cost per sustained responder ratio in the ustekinumab and ixekizumab subgroups was €25,425 and €24,902 respectively. Patients treated with secukinumab had the highest sustained response rate (86%) followed by ixekizumab (80%), ustekinumab (78%), and adalimumab originator (60%).

## Discussion

Results of the CANOVA study showed that biologic therapies were effective for the treatment of moderate-to-severe plaque psoriasis in the real-world setting and confirmed that biologic therapy is an important asset for patients with moderate-to-severe disease. In most patients, the effect of treatment was observed early in time; PASI 75 response rates were remarkable already at week 16 (85.93%) and kept increasing at week 24 (90.26%) up to week 52 (91.47%). A similar trend was observed for the overall PASI 90 responder rates, which progressively increased from 58.57% at week 16, to 73.76% at week 24 and to 75.19% at week 52. Disease remission, measured with PASI 100, was achieved by approximately half of the patients during the study.

From an economic perspective, the study allowed: i) quantification of the economic burden and analysis of the utilization of healthcare resources; ii) assessment of the cost per responder ratios, as an indicator of cost-effectiveness of biologic therapies. The economic analyses were carried out for the overall cohort of patients and for the biologic therapy subgroups that accounted for at least 10% of the sample size within the CANOVA study.

The annual economic impact of plaque psoriasis was about €15,000 per patient, with some variability driven by the different acquisition costs of biologics. This estimate is aligned with relatively recent cost of illness analyses in plaque psoriasis [37, 38]. In our study, annualised total costs in the subgroup of patients receiving secukinumab (€14,892) were in line with the overall group (€15,001). This was somehow expected, as secukinumab was the most used drug in the CANOVA study (41% of patients; *N* = 274 out of *N* = 669). However, the difference became more evident by comparing the ustekinumab (€15,842), and the ixekizumab (€17,858) subgroups with the group of secukinumab treated patients. Adalimumab originator was the cheapest biologic drug among the considered treatments (€13,255) because of its lower acquisition costs, in comparison to other therapies. In fact, cost of biologics was the cost driver, accounting for about 98% of total costs in all subgroups. Secukinumab was the most frequently used biologic drug, followed by ustekinumab, tumor necrosis factor (TNF) inhibitors, and ixekizumab, a ranking that roughly corresponds to the prescription trends for moderate-to-severe plaque psoriasis in Italy [39]. Moreover, during the CANOVA study period, biosimilars were not widely used due to their recent market introduction.

This distribution of costs, by type of resource, was in line with recent literature [40]. The distribution of costs by type confirmed another well-known aspect of plaque psoriasis, which is in common with other autoimmune, inflammatory diseases (e.g. rheumatoid arthritis, ankylosing spondylitis, etc.): management of these conditions is almost exclusively carried out by specialised physicians, who monitor their patients on a regular basis to assess clinical response to pharmacological treatments and prescribe typical routine tests to evaluate the course of the disease (e.g. inflammation markers, renal and liver functions, imaging). Patients rarely consulted general practitioners, and they never required acute care except for rare, unexpected situations (e.g. acute disease progression, treatment-related adverse events).

In the CANOVA protocol, the cost per response was selected as cost-effectiveness indicator, instead of the typical incremental cost-utility ratio used in standard cost-effectiveness analyses [41, 42]. Although less common than ICER, this indicator has already been used in the past [43]. The choice of cost per response analysis was driven by a number of factors intrinsically correlated to the design of the CANOVA study: i) the objective of the study was to provide a picture of the economic impact of biologic treatments, and not to conduct 1:1 pharmacoeconomic comparisons between biologics; ii) a traditional cost-utility approach was not pursued because of the CANOVA study design, consisting of a mainly retrospective phase, during which patient utilities (required in a cost per QALY assessment) could not be collected; iii) a cost-utility assessment would be a valid approach in a chronic condition like plaque psoriasis when a longer time horizon (of at least 10 years) can be observed, which is not the case of the CANOVA study. The cost per response is an informative indicator because, in principle, it allows detecting treatment groups with the lowest cost per therapeutic success and ranking them according to economic opportunity.

In all the analyses, adalimumab originator and secukinumab had the lowest and second-lowest cost per responder ratio, respectively, with a slight difference between the two. This difference was driven by the fact thatsecukinumab requires an induction phase of 4 weeks (5 secukinumab administration), while adalimumab does not. Afterwards, during maintenance, the cost of the two drugs becomes similar. However, the slightly higher costs of secukinumab were offset by an earlier and more sustained response, compared with adalimumab. Unlike adalimumab originator and secukinumab, it was not possible to rank cost per responder ratio of the overall group, and of the the ustekinumab and ixekizumab subgroups. As a matter of fact, the lowest cost per responder ratio among these three groups changed based on type of indicator (PASI 75, PASI 90, PASI 100) and time of assessment (16 weeks, 24 weeks, 52 weeks).

Finally, an interesting trend was found, concerning the cost per sustained response. PASI 75 response rates at week 52 of > 85% were observed in all different treatment groups, suggesting that in a 1-year timeframe, biologics guarantee remarkable clinical benefit in most patients. However, time to response seemed to depend on treatment, with earlier response rates being observed in the secukinumab subgroup, followed by the ixekizumab, ustekinumab, and finally the adalimumab originator subgroup. Therefore, the post-hoc analysis of the cost per sustained response (PASI 75) showed some favourable trends for secukinumab, driven by its high response rate, which was achieved early in time (16 weeks) and confirmed subsequently (24 and 52 weeks) in comparison with ixekizumab and adalimumab originator. At 16 and 24 weeks, secukinumab was associated with higher response rates than ustekinumab; at 52 weeks, the response rate became similar in the two subgroups of patients (secukinumab: 93%,ustekinumab: 94%). The findings of this economic analysis should be evaluated taking into consideration potential limitations. First of all, it is likely that both annual direct healthcare costs of the disease and cost per response ratios might have been overestimated. In fact, the cost of pharmacological treatment, which was the cost driver in this analysis, was calculated using ex-manufacturer unit prices, extracted from the databases of the Italian Drug Agency (AIFA). Indeed, the price of these therapies might be lower in the real world, as manufacturers might grant discounts during the procurement process. Also, costs of therapies after procurement might vary by region, and even by hospital, depending on local purchase mechanisms. However, since the amount of such discounts is not clearly known (rarely in the public domain), the use of official prices to conduct the analysis was preferred. Secondly, one could argue that only direct costs of the disease were captured, but it is well known in literature that plaque psoriasis (as well as other autoimmune conditions) poses a significant economic burden on patients’ productivity [38]. Thirdly, at the date of the study, biosimilars were little used due to their recent entry into the market. However, their entry into the market could change the study results. For this reason, an update of the present analysis would be desirable, when more robust data are available, coming from real-world use.

Furthermore, given that the present analysis assessed the cost of disease by treatment groups, another potential limitation is that costs were not adjusted by patient characteristics at baseline. Plausibly, there could be heterogeneity in patients’ characteristics (in terms of comorbidities, health conditions) that might affect selection of the appropriate therapy and hence costs and response rates [19, 20]. However, due to the exploratory purpose of the study (comparison by treatment groups was not a primary objective), the authors believe that results of the analysis remain valid and informative.

Finally, in the assessment of clinical response at week 16, 24, and 52, the number of patients evaluated could be variable at different time points. However, the calculation methodology adopted was designed to avoid potential selection bias, also including patients who discontinued treatment.

Despite those limitations, we still believe that the analysis has great value in informing budget holders on the costs sustained by the Italian National Health System (SSN), and on consequent budget allocation. Furthermore, there might have been some differences in patients’ characteristics by treatment group, somehow affecting the results of the stratified analyses. As a comment to this methodological issue, it can be said that: i) most of the potential confounding factors (time since diagnosis, number of prior lines received, proportion of patients switching to a new biologic therapy) were well balanced by treatment group; ii) small treatment subgroups were excluded from stratified analysis to avoid issues of consistency; iii) the economic analysis was not designed to compare therapies among each other from an economic perspective, or to test hypotheses of cost-effectiveness superiority of one therapy versus the others, but rather to describe costs and cost per response, and eventually detect trends requiring further investigation in the future. For all these reasons, and despite some possible limitations, this concomitant assessment of treatment costs against the expected therapeutic response remains a valuable tool providing physicians and payers with additional useful insights which can complement the traditional risk-benefit profile assessment and drive treatment decisions.

## Conclusion

We acknowledge that the choice of the appropriate therapy in a condition like plaque psoriasis is driven by complex and individualised considerations, which would include intrinsic patient characteristics that cannot be easily incorporated in a simple “cost-effectiveness equation”. However, in the absence of long-term data showing clear superiority of one drug versus the others, time to response and then cost per response could provide payers and prescribers with valuable information to inform on treatment decision-making.

## Supplementary Information

**Additional file 1.**

## Data Availability

The datasets used and/or analysed during the current study available from the corresponding author on reasonable request.

## Abbreviations

BSA: Body Surface Area
CANOVA: EffeCtiveness of biologic treAtmeNts for plaque psOriasis in Italy: an obserVAtional longitudinal study of real-life clinical practice
DRG: Diagnosis Related Group
ED: Emergency Department
GP: General Practitioner
IQR: InterQuartile Range
NHS: National Healthcare Service
PASI: Psoriasis Area and Severity Index score
QoL: Quality of Life
SSN: Servizio Sanitario Nazionale (national healthcare service in Italian)

## References

[1] Rapp SR, Feldman SR, Exum ML, Fleischer AB, Reboussin DM (1999). Psoriasis causes as much disability as other major medical diseases. J Am Acad Dermatol.

[2] World Health Organization. Global report on psoriasis. World Health Organization. 2016 [cited 2020 Oct 19]. Available from: www.who.int

[3] Takeshita J, Grewal S, Langan SM, Mehta NN, Ogdie A, Van Voorhees AS, et al. Psoriasis and comorbid diseases: Epidemiology. Vol. 76, Journal of the American Academy of Dermatology. Mosby Inc.; 2017. p. 377–90. 10.1016/j.jaad.2016.07.064 PMC5731650 28212759

[4] Gupta MA, Gupta AK. Depression and suicidal ideation in dermatology patients with acne, alopecia areata, atopic dermatitis and psoriasis. Br J Dermatol. 1998;139(5):846–50. [cited 2020 Oct 19] Available from: https://pubmed.ncbi.nlm.nih.gov/9892952/10.1046/j.1365-2133.1998.02511.x 9892952

[5] Schmitt J, Ford DE. Understanding the relationship between objective disease severity, psoriatic symptoms, illness-related stress, health-related quality of life and depressive symptoms in patients with psoriasis - a structural equations modeling approach. Gen Hosp Psychiatry. 2007;29(2):134–40. [cited 2020 Oct 19] Available from: https://pubmed.ncbi.nlm.nih.gov/17336662/10.1016/j.genhosppsych.2006.12.004 17336662

[6] Hayes J, Koo J. Psoriasis: Depression, anxiety, smoking, and drinking habits. Dermatol Ther. 2010;23(2):174–80. [cited 2020 Oct 19] Available from: https://pubmed.ncbi.nlm.nih.gov/20415825/10.1111/j.1529-8019.2010.01312.x 20415825

[7] Jagtiani A, Nishal P, Jangid P, Sethi S, Dayal S, Kapoor A. Depression and suicidal ideation in patients with acne, psoriasis, and alopecia areata. J Ment Heal Hum Behav. 2017 22(1):50. [cited 2020 Oct 19] Available from: http://www.jmhhb.org/text.asp?2017/22/1/50/210700

[8] Gisondi P, Altomare G, Ayala F, Bardazzi F, Bianchi L, Chiricozzi A, et al. Italian guidelines on the systemic treatments of moderate-to-severe plaque psoriasis. J Eur Acad Dermatology Venereol. 2017;31(5):774–90. [cited 2020 Oct 19] Available from: https://pubmed.ncbi.nlm.nih.gov/28244153/10.1111/jdv.14114 28244153

[9] Vena GA, Altomare G, Ayala F, Berardesca E, Calzavara-Pinton P, Chimenti S, et al. Incidence of psoriasis and association with comorbidities in Italy: A 5-year observational study from a national primary care database. Eur J Dermatology. 2010;20(5):593–8. [cited 2020 Oct 19] Available from: http://www.jle.com/fr/revues/ejd/e-docs/incidence_of_psoriasis_and_association_with_comorbidities_in_italy_a_5_year_observational_study_from_a_national_primary_care_d_285881/article.phtml?tab=texte 10.1684/ejd.2010.1017 20605768

[10] Prignano F, Rogai V, Cavallucci E, Bitossi A, Hammen V, Cantini F. Epidemiology of Psoriasis and Psoriatic Arthritis in Italy—a Systematic Review. Curr Rheumatol Rep. 2018 20(7). [cited 2020 Oct 23] Available from: https://pubmed.ncbi.nlm.nih.gov/29846817/10.1007/s11926-018-0753-1 29846817

[11] Colombo GL, Altomare GF, Peris K, Martini P, Quarta G, Congedo M, et al. Moderate and severe plaque psoriasis: cost-of-illness study in Italy. Vol. 4, Therapeutics and Clinical Risk Management. 2008. 10.2147/tcrm.s2740 PMC2504078 18728854

[12] Lasagni C, Bigi L, Conti A, Pellacani G. Successful therapy of plaque-type psoriasis with secukinumab in patients with multiple comorbidities treated with previous biologic therapies. J Dermatolog Treat. 2018;29(sup2):5–8. [cited 2020 Oct 19] Available from: https://pubmed.ncbi.nlm.nih.gov/30403898/10.1080/09546634.2018.1543843 30403898

[13] Gisondi P, Altomare G, Ayala F, Bardazzi F, Bianchi L, Chiricozzi A, et al. Italian guidelines on the systemic treatments of moderate-to-severe plaque psoriasis. J Eur Acad Dermatology Venereol. 2017;31(5):774–90. [cited 2020 Oct 20] Available from: https://pubmed.ncbi.nlm.nih.gov/28244153/10.1111/jdv.14114 28244153

[14] Greiner RA, Braathen LR. Cost-effectiveness of biologics for moderate-to-severe psoriasis from the perspective of the Swiss healthcare system. Eur J Dermatology. 2009;19(5):494–9. [cited 2020 Oct 20] Available from: https://pubmed.ncbi.nlm.nih.gov/19502153/10.1684/ejd.2009.0725 19502153

[15] Mansouri B, Patel M, Menter A. Biological therapies for psoriasis. Vol. 13, Expert Opinion on Biological Therapy. Expert Opin Biol Ther; 2013; 1715–30. [cited 2020 Oct 20] Available from: https://pubmed.ncbi.nlm.nih.gov/24160990/10.1517/14712598.2013.853739 24160990

[16] Chi CC, Wang SH. Efficacy and cost-efficacy of biologic therapies for moderate to severe psoriasis: A meta-analysis and cost-efficacy analysis using the intention-to-treat principle. Biomed Res Int. 2014;2014. [cited 2020 Oct 20] Available from: https://pubmed.ncbi.nlm.nih.gov/24605338/10.1155/2014/862851 PMC3925556 24605338

[17] Blauvelt A, Prinz JC, Gottlieb AB, Kingo K, Sofen H, Ruer-Mulard M, et al. Secukinumab administration by pre-filled syringe: Efficacy, safety and usability results from a randomized controlled trial in psoriasis (FEATURE). Br J Dermatol. 2015;172(2):484–93. [cited 2020 Oct 20]Available from: https://pubmed.ncbi.nlm.nih.gov/25132411/10.1111/bjd.13348 25132411

[18] Paul C, Lacour JP, Tedremets L, Kreutzer K, Jazayeri S, Adams S, et al. Efficacy, safety and usability of secukinumab administration by autoinjector/pen in psoriasis: A randomized, controlled trial (JUNCTURE). J Eur Acad Dermatology Venereol. 2015;29(6):1082–90. [cited 2020 Oct 20] Available from: https://pubmed.ncbi.nlm.nih.gov/25243910/10.1111/jdv.12751 25243910

[19] Kaushik SB, Lebwohl MG. Psoriasis: which therapy for which patient: focus on special populations and chronic infections. J Am Acad Dermatol. 2019;80(1):43–53. Available from: 10.1016/j.jaad.2018.06.056, 2019. 10.1016/j.jaad.2018.06.056 30017706

[20] Kaushik SB, Lebwohl MG. Psoriasis: which therapy for which patient: psoriasis comorbidities and preferred systemic agents. J Am Acad Dermatol. 2019;80(1):27–40. Available from: 10.1016/j.jaad.2018.06.057, 2019. 10.1016/j.jaad.2018.06.057 30017705

[21] Kragballe K, Van De Kerkhof PCM, Gordon KB. Unmet needs in the treatment of psoriasis. Vol. 24, European Journal of Dermatology. John Libbey Eurotext; 2014;523–32. [cited 2020 Oct 20] Available from: https://pubmed.ncbi.nlm.nih.gov/25115238/10.1684/ejd.2014.2403 25115238

[22] Ahn CS, Gustafson CJ, Sandoval LF, Davis SA, Feldman SR. Cost effectiveness of biologic therapies for plaque psoriasis. Am J Clin Dermatol. 2013;14(4):315–26. [cited 2020 Oct 20] Available from: https://pubmed.ncbi.nlm.nih.gov/23696234/10.1007/s40257-013-0030-z 23696234

[23] Makady A, Ham R ten, de Boer A, Hillege H, Klungel O, Goettsch W. Policies for Use of Real-World Data in Health Technology Assessment (HTA): A Comparative Study of Six HTA Agencies. Value Heal. 2017;20(4):520–32. [cited 2020 Sep 18]Available from: https://pubmed.ncbi.nlm.nih.gov/28407993/10.1016/j.jval.2016.12.003 28407993

[24] Berger ML, Sox H, Willke RJ, Brixner DL, Eichler HG, Goettsch W, et al. Good practices for real-world data studies of treatment and/or comparative effectiveness: Recommendations from the joint ISPOR-ISPE Special Task Force on real-world evidence in health care decision making. Pharmacoepidemiol Drug Saf. 2017;26(9):1033–9. [cited 2020 Oct 19] Available from: https://pubmed.ncbi.nlm.nih.gov/28913966/10.1002/pds.4297 PMC5639372 28913966

[25] Schaefer CP, Cappelleri JC, Cheng R, Cole JC, Guenthner S, Fowler J, et al. Health care resource use, productivity, and costs among patients with moderate to severe plaque psoriasis in the United States. J Am Acad Dermatol. 2015;73(4):585–593.e3. 10.1016/j.jaad.2015.06.049 26253364

[26] Feldman S, Burudpakdee C, Gala S, Mallya U. Systematic Literature Review of Economic Burden of Chronic Plaque Psoriasis. 2013. 10.1586/14737167.2014.933671 25052261

[27] Colombo D, Bianchi L, Fabbrocini G, Corrao S, Offidani A, Stingeni L, et al. Real-world evidence of biologic treatments in moderate-severe psoriasis in Italy: Results of the CANOVA (EffeCtiveness of biologic treAtmeNts for plaque psOriasis in Italy: an obserVAtional longitudinal study of real-life clinical practice) study. - Unpub. 2020; 10.1111/dth.15166 34676662

[28] Fredriksson T, Pettersson U. Severe psoriasis — Oral therapy with a new retinoid. Dermatology. 1978;157(4):238–44. [cited 2020 Oct 20] Available from: https://pubmed.ncbi.nlm.nih.gov/357213/10.1159/000250839 357213

[29] Feldman SR, Krueger GG. Psoriasis assessment tools in clinical trials. In: Annals of the Rheumatic Diseases. Ann Rheum Dis; 2005 [cited 2020 Oct 20]. Available from: https://pubmed.ncbi.nlm.nih.gov/15708941/10.1136/ard.2004.031237 PMC1766877 15708941

[30] Navarini AA, Poulin Y, Menter A, Gu Y, Teixeira HD. Analysis of body regions and components of PASI scores during adalimumab or methotrexate treatment for patients with moderate-to-severe psoriasis - PubMed. J Drugs Dermatol. 2014;13(5):554–62. [cited 2020 Oct 20] Available from: https://pubmed.ncbi.nlm.nih.gov/24809878/24809878

[31] Mrowietz U, Kragballe K, Reich K, Spuls P, Griffiths CEM, Nast A, et al. Definition of treatment goals for moderate to severe psoriasis: A European consensus. Arch Dermatol Res. 2011;303(1):1–10. [cited 2020 Oct 20]Available from: https://pubmed.ncbi.nlm.nih.gov/20857129/10.1007/s00403-010-1080-1 PMC3016217 20857129

[32] Italian Ministry of Health (2013a). Outpatient intervention tariffs. Italian Republic Official Gazette Chapter n 23; Supplement n 8; 28 January 2013.

[33] Italian Ministry of Health (2013a). Inpatient intervention tariffs. Italian Republic Official Gazette. Chapter n. 23; Supplement n. 8; 28 January 2013.

[34] Garattini. Duration and costs of general practitioners’ visits: the DYSCO project [Italian article]. Farmeconomia e percorsi terapeutici. 2003. 4 (2). Farmeconomia e percorsi Ter. 2003;4(2):109–14.

[35] National Institute of Statistics of Italy (ISTAT). Inflation rates. Available at: http://rivaluta.istat.it/Rivaluta/. Accessed: September 2018.

[36] Ministero della salute. Tariffe delle prestazioni ospedaliere. Supplemento n.8, Gazzetta Ufficiale n. 23, 2013. Allegato 1.

[37] Spandonaro F, Ayala F, Berardesca E, Chimenti S, Girolomoni G, Martini P, et al. The cost effectiveness of biologic therapy for the treatment of chronic plaque psoriasis in real practice settings in ITALY. BioDrugs. 2014;28(3):285–95. [cited 2020 Oct 23] Available from: https://pubmed.ncbi.nlm.nih.gov/24567261/10.1007/s40259-014-0084-3 PMC4030097 24567261

[38] Polistena B, Calzavara-Pinton P, Altomare G, Berardesca E, Girolomoni G, Martini P, et al. The impact of biologic therapy in chronic plaque psoriasis from a societal perspective: An analysis based on Italian actual clinical practice. J Eur Acad Dermatology Venereol. 2015;29(12):2411–6. [cited 2020 Oct 23] Available from: https://pubmed.ncbi.nlm.nih.gov/26370321/10.1111/jdv.13307 26370321

[39] Colombo GL, Di Matteo S, Martinotti C, Jugl SM, Gunda P, Naclerio M, et al. Budget impact model of secukinumab for the treatment of moderate-to-severe psoriasis, psoriatic arthritis, and ankylosing spondylitis in Italy: A cross-indication initiative. Clin Outcomes Res. 2018;10:477–91. [cited 2021 May 17] Available from: /pmc/articles/PMC6121773/. 10.2147/CEOR.S171560 PMC6121773 30214261

[40] Guerriero F, Orlando V, Monetti VM, Russo V, Menditto E. Biological therapy utilization, switching, and cost among patients with psoriasis: Retrospective analysis of administrative databases in southern Italy. Clin Outcomes Res. 2017;9:741–8. [cited 2020 Oct 23] Available from: /pmc/articles/PMC5716306/?report=abstract. 10.2147/CEOR.S147558 PMC5716306 29238210

[41] Küster D, Nast A, Gerdes S, Weberschock T, Wozel G, Gutknecht M, et al. Cost-effectiveness of systemic treatments for moderate-to-severe psoriasis in the German health care setting. Arch Dermatol Res. 2016; 10.1007/s00403-016-1634-y 26961372

[42] Hendrix N, Ollendorf DA, Chapman RH, Loos A, Liu S, Kumar V, et al. Cost-effectiveness of targeted pharmacotherapy for moderate to severe plaque psoriasis. J Manag Care Spec Pharm. 2018; 10.18553/jmcp.2018.24.12.1210 PMC10398188 30479197

[43] Ahn CS, Gustafson CJ, Sandoval LF, Davis SA, Feldman SR. Cost effectiveness of biologic therapies for plaque psoriasis. Am J Clin Dermatol. 2013;14(4):315–26. [cited 2020 Sep 18] Available from: https://pubmed.ncbi.nlm.nih.gov/23696234/10.1007/s40257-013-0030-z 23696234

