# Supplementary material for: A real-world economic analysis of biologic therapies for moderate-to-severe plaque psoriasis in Italy: results of the CANOVA observational longitudinal study
Source: BMC Health Serv Res. 2021 Sep 6;21:924. doi: 10.1186/s12913-021-06866-7 (PMC8422702; doi:10.1186/s12913-021-06866-7)
Supplement: Supplementary file 1 — Additional file 1. [file 12913_2021_6866_MOESM1_ESM.docx]

# Supplementary Information

**Additional file 1: Figure A-1 Patient’s scheme**

**
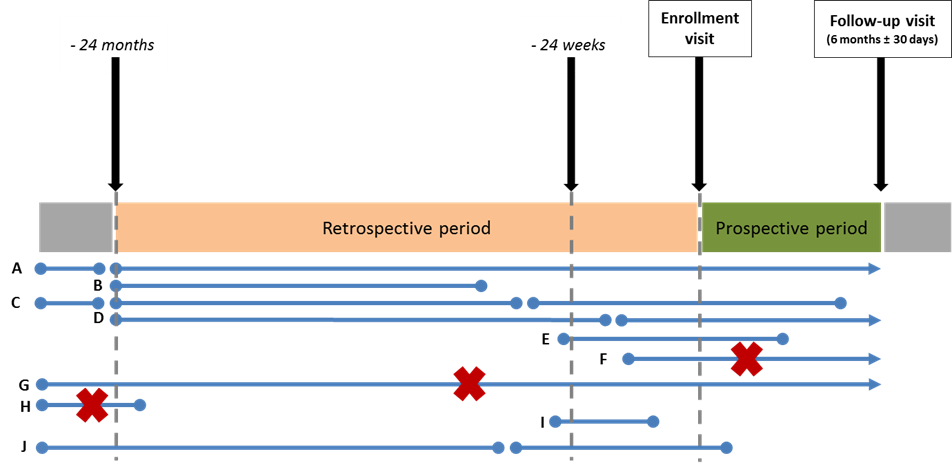
**

*Each letter represents one patient, and each blue line represents a biologic therapy line; if the line ends with a circle then the treatment line was interrupted, while if the line ends with an arrow then the treatment line was not interrupted. All patients from “A” to “E” were eligible for the study because in all these cases at least one line of biologic therapy for psoriasis was initiated at least 24 weeks before enrolment visit but no more than 24 months before enrolment visit; patient “B” is also eligible, even though he/she has interrupted the treatment line before enrollment. Regarding patients “A” and “C”, only the biologic therapy lines initiated within the retrospective period were considered (e.g. for patient “C”, only data regarding 2^nd^ and 3^rd^ line were collected). Patients with a red cross (i.e. “F”, “G”, and “H”) were not eligible because in all these cases every line of biologic therapy for psoriasis was initiated either less than 24 weeks before enrolment visit or more than 24 months before enrolment visit. Patient “I” is eligible because in this case at least one line of biologic therapy for psoriasis was initiated at least 24 weeks but no more than 24 months before enrolment visit. Ppatient “J” is also eligible, but only the 2^nd^ therapy line was considered (because the first therapy line did not start within the retrospective period).*
